# A Rare Case of a Schwannoma on the Foot – Case Report

**DOI:** 10.1055/s-0042-1756150

**Published:** 2022-09-28

**Authors:** José Carlos Sousa Miradouro, Tiago Costa, Nuno Silva, João Afonso

**Affiliations:** 1 Centro Hospitalar do Tâmega e Sousa, Porto, Portugal.

**Keywords:** foot, nerve sheath neoplasms, peroneal nerve, schwannoma

## Abstract

A Schwannoma is an infrequent lesion. This tumor derives from the myelin sheath of the peripheral nerves; in most cases, it is benign and rarely presents in the foot and ankle region. Patients affected by this type of pathology are usually asymptomatic. Still, they sometimes have sensory or motor neurologic symptoms if the tumor is large enough to cause direct or indirect compression of the affected nerve. A 55-year-old male patient presented to our department with non-traumatic swelling and pain in the lateral aspect of the right foot and leg. A magnetic resonance imaging (MRI) scan of the right leg revealed a well-circumscribed lesion, measuring 2,5 by 1 cm, showing hypointensity on T1 sequences and hyperintensity on T2, compatible with a superficial peroneal nerve sheath cells tumor. Surgical excision of the lesion was performed, and the histopathological examination confirmed the initial suspicion—Schwannoma of the superficial peroneal nerve. The postoperative period was uneventful, with progressive improvement of pain and complete functional recovery without neurological deficits. Rigorous clinical examination associated to MRI scans allow adequate diagnosis as well as the exclusion of other pathologies with similar clinical presentation. Thus, the surgeon has to be aware of all the data for an effective diagnosis and treatment in this type of rare pathology that cannot be neglected.

## Introduction

Schwannomas are an infrequent lesion. This tumor derives from the myelin sheath of the peripheral nerves; in most cases, it is benign. It occurs mostly among patients between the third and fifth decades of life, and has a homogeneous distribution between females and males, as well as among different ethnicities. 1

It is a soft tissue tumor that is rarely located in the foot and ankle, preferentially located in anatomical regions such as the head, neck, trunk, and upper limbs. 2 Patients affected by this type of pathology are, in the vast majority of cases, asymptomatic. Still, there may sometimes be sensory or motor neurologic symptoms if the tumor is large enough to cause direct or indirect compression of the affected nerve. 3

## Case Report

A 55-year-old male patient presented to us with a complaint of 2 months of mild pain located on the lateral aspect of the right foot and ankle, in the absence of associated trauma. Clinical examination revealed a firm mass with approximately 3 by 1 cm, on the lateral and distal surface of the right leg, with positive Tinel's sign on percussion and no neurological deficits.

**Fig. 1:**
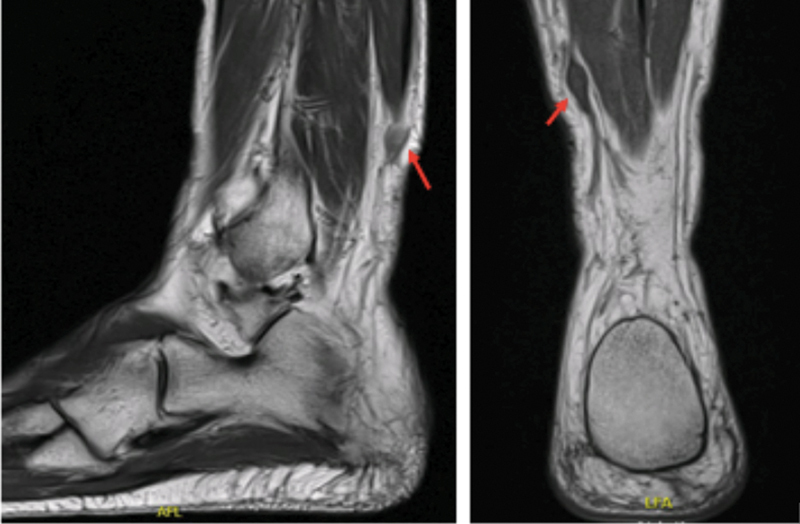
Magnetic resonance imaging (MRI) scan revealed hypointensity on T1.

**Fig. 2:**
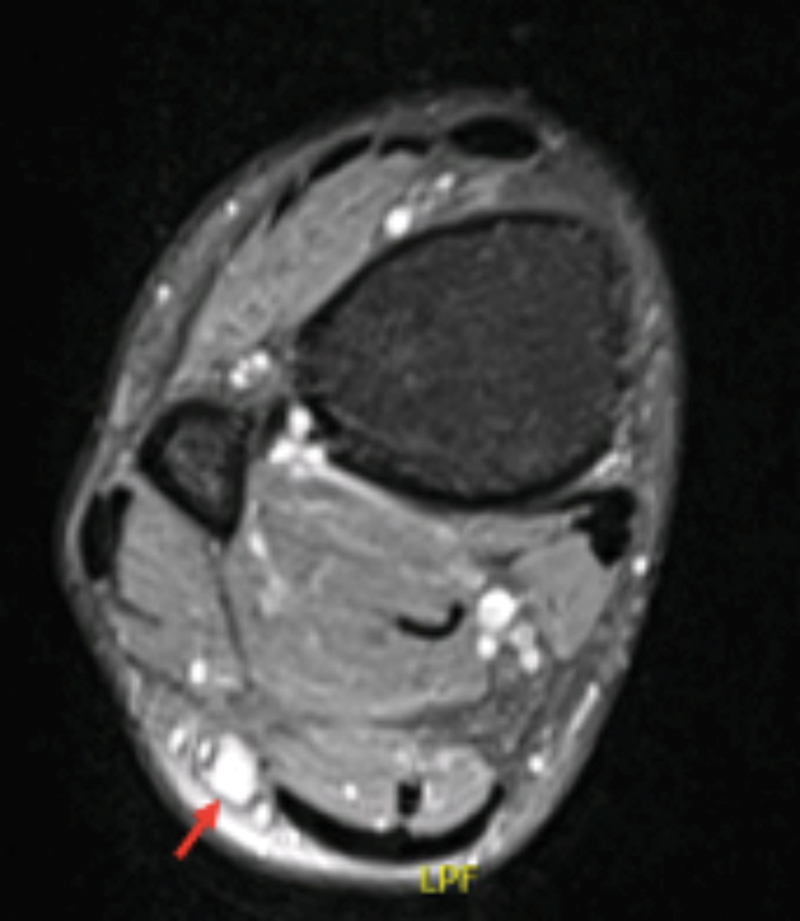
Magnetic resonance imaging (MRI) scan revealed hypeintensity on T2.

Magnetic resonance imaging (MRI) scan of the right leg revealed a well-circumscribed lesion, measuring 2,5 by 1 cm, showing hypointensity on T1 sequences ( Fig. 1 ) and hyperintensity on T2 ( Fig. 2 ), compatible with a superficial peroneal nerve sheath cells tumor.

**Fig. 3:**
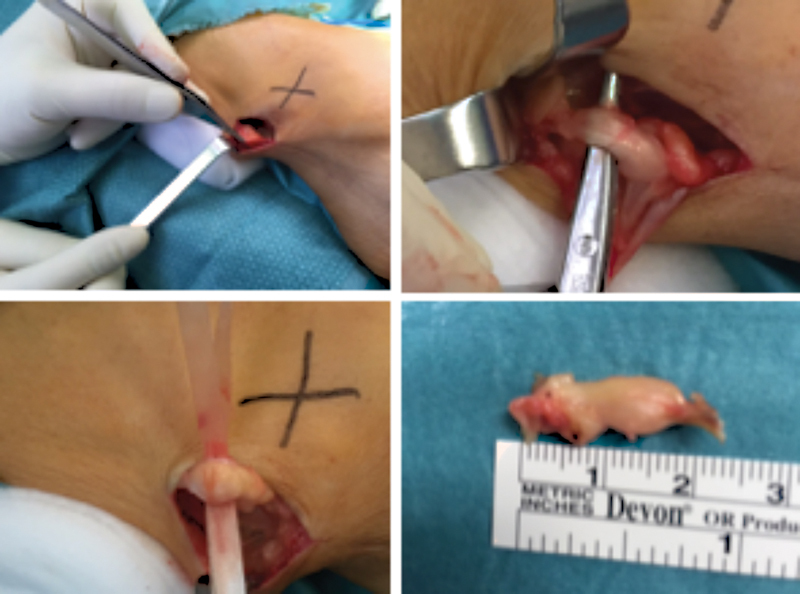
Excised tumor.

Afterwards, surgical excision of the lesion was conducted using the enucleation technique, with an anterolateral approach, exposing the tumor, followed by a perineural dissection and tumoral detachment of the nerve sheath ( Fig. 3 ). After further inspection at the end of the procedure, we concluded that the tumor was removed properly and without compromising the structure of the closest vessels and nerves.

The excised tumor was sent for a histological study which confirmed the initial suspicion—Schwannoma of the superficial peroneal nerve. Hematoxylin and eosin stain revealed the characteristic hypercellular (Antoni A) and hypocellular (Antoni B) regions of the specimen.

The postoperative period was uneventful, with progressive improvement of pain and complete functional recovery, without neurological deficits.

We currently have a 24-month follow-up, and the patient has no major complaints.

## Discussion

Peripheral nerve sheath tumors, such as Schwannomas, are infrequent in the anatomical region of the foot and ankle, accounting for approximately 4 to 10% of all soft tissue tumors in this region, depending on the series. 4 5

The authors present an unusual case of a Schwannoma affecting the lower limb. There are very sparse case reports of this tumor of the superficial peroneal nerve described in literature. Schwannomas are typically benign and indolent, and are often diagnosed incidentally due to its asymptomatic presentation. However, in a few cases it can cause localized pain and paresthesia due to compression of the surrounding nerves. Evolution to malignancy is rare, but it is of paramount importance to distinguish Schwannoma from a malignant peripheral nerve sheath tumor, though it is not always easy. The use of MRI can help to better characterize these tumors, showing hyperintense signals on T2-weighted images and isointense signals on T1-weighted images. 6 7

Clinical examination, MRI, and surgical excision with histological evaluation can confirm the diagnosis, as well as exclude the presence of a malignant tumor. In a series by Carvajal et al., these methods allowed for accurate diagnosis in 14% of the cases. 4

Schwannomas are well encapsulated, eventually causing displacement of nerve fascicles. For that reason, it is generally believed that enucleation from the nerve can be approached without producing neurological deficits. 8

Surgical excision or enucleation is the gold standard when it comes to treatment, though, for smaller tumors, watchful waiting can be an option. Extreme care is vital regarding tumor dissection of the associated nerves to preserve or restore nerve function to the maximum level. Recurrence is extremely rare (less than 1%) unless tumor tissue is removed improperly. 5 8 9

Given that the symptoms in this type of lesion are often mild or may not even exist, the delay in diagnosis is frequent in cases of lower limb schwannoma, and there are even reports of cases where it took approximately 15 years to get the proper diagnosis. Therefore, rigorous clinical examination, associated with MRI scans and histological evaluation, allow for adequate diagnosis and the exclusion of other pathologies with similar clinical presentation. 10

Thus, the surgeon has to be aware of all the data for an effective diagnosis and treatment in this type of rare pathology that cannot be neglected.
