# Supplementary material for: A Rare Case of a Schwannoma on the Foot – Case Report
Source: Rev Bras Ortop (Sao Paulo). 2022 Sep 28;59(Suppl 1):e88–90. doi: 10.1055/s-0042-1756150 (PMC11254422; doi:10.1055/s-0042-1756150)
Supplement: Related texts

# Research article: Caso raro de um schwannoma no pé – Relato de caso

**Keywords:** neoplasias da bainha neural, nervo fibular, pé, schwanoma

## Abstract

Um schwannoma é uma lesão pouco frequente, sendo um tumor que deriva da bainha de mielina dos nervos periféricos; na maioria dos casos, este tumor é benigno e raramente se apresenta na região do pé e tornozelo. Os pacientes afetados por esse tipo de patologia são geralmente assintomáticos. Ainda assim, às vezes eles têm sintomas neurológicos sensoriais ou motores se o tumor for grande o suficiente para causar a compressão direta ou indireta do nervo afetado. Um paciente do gênero masculino de 55 anos se apresentou ao nosso departamento com inchaço não traumático e dor no aspecto lateral do pé direito e da perna. A ressonância magnética (RM) da perna direita revelou uma lesão bem-circunscrita, medindo 2,5 por 1 cm, mostrando hipointensidade nas sequências T1 e hiperintensidade em T2, compatível com um tumor superficial de células do nervo peronal. Foi realizada excisão cirúrgica da lesão e o exame histopatológico confirmou a suspeita inicial – Schwannoma do nervo peroneal superficial. O pós-operatório foi tranquilo, com melhora progressiva da dor e recuperação funcional completa sem déficits neurológicos. Exame clínico rigoroso associado aos exames de RM permitem diagnóstico adequado, bem como a exclusão de outras patologias com apresentação clínica semelhante. Assim, o cirurgião tem que estar atento a todos os dados para um diagnóstico e tratamento eficazes nesse tipo de patologia rara que não pode ser negligenciada.

## Introdução

Schwannomas são uma lesão pouco frequente. Esse tipo de tumor deriva da bainha de mielina dos nervos periféricos e, na maioria dos casos, é benigno. Ocorre principalmente entre pacientes entre a terceira e quinta décadas de vida, e possui uma distribuição homogênea entre mulheres e homens, assim como entre diferentes etnias. 1

É um tumor de tecido mole que raramente se localiza no pé e tornozelo, estando preferencialmente em regiões anatômicas como cabeça, pescoço, tronco e membros superiores. 2 Os pacientes afetados por esse tipo de patologia são, na grande maioria dos casos, assintomáticos. Ainda assim, podem ocorrer sintomas neurológicos sensoriais ou motores se o tumor for grande o suficiente para causar compressão direta ou indireta do nervo afetado. 3

## Relato de caso

Um paciente do gênero masculino de 55 anos apresentou-nos queixa de dois meses de dor leve localizada no aspecto lateral do pé direito e tornozelo, na ausência de trauma associado. O exame clínico revelou uma massa firme com aproximadamente 3 por 1 cm, na superfície lateral e distal da perna direita, com sinal positivo de Tinel na percussão e sem déficits neurológicos.

A ressonância magnética (RM) da perna direita revelou uma lesão bem circunscrita, medindo 2,5 por 1 cm, mostrando hipointensidade nas sequências T1 ( Fig. 1 ) e hiperintensidade em T2 ( Fig. 2 ), compatível com um tumor superficial de células de bainha nervosa peroneal.

**Fig. 1:** A ressonância magnética (RM) revelou hipointensidade no T1.

**Fig. 2:** A ressonância magnética (RM) revelou hiperintensidade no T2.

Posteriormente, foi realizada a excisão cirúrgica da lesão utilizando técnica de enucleação, por abordagem anterolateral, expondo o tumor, seguida de dissecção perineural e descolamento tumoral da bainha nervosa ( Fig. 3 ). Após nova inspeção no final do procedimento, concluiu-se que o tumor foi removido adequadamente e sem comprometer a estrutura dos vasos e nervos mais próximos.

**Fig. 3:** Tumor excisado.

O tumor excisado foi enviado para um estudo histológico que confirmou a suspeita inicial – Schwannoma do nervo peroneal superficial. A hematoxilina e a mancha de eosina revelaram as regiões características hipercelulares (Antoni A) e hipocelulares (Antoni B) do espécime.

O pós-operatório foi tranquilo, com melhora progressiva da dor e recuperação funcional completa sem déficits neurológicos.

Atualmente temos um acompanhamento de 24 meses e o paciente não tem queixas graves.

## Discussão

Tumores periféricos de bainha nervosa, como schwannomas, são pouco frequentes na região anatômica do pé e tornozelo, representando aproximadamente 4 a 10% de todos os tumores de tecido mole nesta região, dependendo da série. 4 5

Os autores apresentam um caso incomum de um Schwannoma afetando o membro inferior. Existem relatos de casos muito esparsos deste tumor do nervo peroneal superficial descritos na literatura.

Schwannomas normalmente são benignos e indolentes, e são frequentemente diagnosticados incidentalmente devido à sua apresentação assintomática. No entanto, em alguns casos pode causar dor localizada e parestesia devido à compressão dos nervos circundantes. A evolução da malignidade é rara, e apesar de nem sempre ser fácil, é de suma importância distinguir Schwannoma de um tumor maligno da bainha do nervo periférico. O uso de RM pode ajudar a caracterizar melhor esses tumores, mostrando sinais hiperintensos em imagens ponderadas em T2 e sinais isointensos em imagens ponderadas por T1. 6 7

O exame clínico, RM e excisão cirúrgica com avaliação histológica permitem confirmar o diagnóstico, além de ajudar a excluir a presença de um tumor maligno. Em uma série por Carvajal et al., estes métodos possibilitaram o diagnóstico preciso de 14% dos casos. 4

Schwannomas estão bem encapsulados, eventualmente causando deslocamento de fascículos nervosos. Por essa razão, acredita-se geralmente que a enucleação do nervo pode ser abordada sem produzir déficits neurológicos. 8

Excisão cirúrgica ou enucleação é o padrão-ouro quando se trata de tratamento, embora para tumores menores, a espera atenta pode ser uma opção. Cuidados extremos são vitais em relação à dissecção tumoral dos nervos associados para preservar ou restaurar a função nervosa ao nível máximo. A recidiva é extremamente rara (menos de 1%) a menos que o tecido tumoral seja removido incorretamente. 5 8 9

Dado que os sintomas nesse tipo de lesão são muitas vezes leves ou podem até não existir, a demora no diagnóstico é frequente em casos de schwannoma de membros inferiores, e há até relatos de casos em que se levou aproximadamente 15 anos para obter o diagnóstico adequado. Portanto, o rigoroso exame clínico associado aos exames de RM e avaliação histológica permitem o diagnóstico adequado, bem como a exclusão de outras patologias com apresentação clínica semelhante. 10

Assim, o cirurgião tem que estar atento a todos os dados para um diagnóstico e tratamento eficazes nesse tipo de patologia rara que não pode ser negligenciada.
